# Scientific collaboration, research funding, and novelty in scientific knowledge

**DOI:** 10.1371/journal.pone.0271678

**Published:** 2022-07-25

**Authors:** Hyunha Shin, Keungoui Kim, Dieter F. Kogler

**Affiliations:** 1 Spatial Dynamics Lab, School of Architecture, Planning & Environmental Policy, University College Dublin, Dublin, Ireland; 2 School of Applied Artificial Intelligence, Handong Global University, Pohang, South Korea; 3 Insight Centre for Data Analytics, University College Dublin, Dublin, Ireland; Indian Institute of Technology Delhi, INDIA

## Abstract

Disruptive advancements in science and technology often rely on new ideas and findings, which in turn brings us to focus on the value of novelty in scholarly activities. Using Web of Science publication data from European regions for the period between 2008 and 2017, this study examines, first, the impact of scientific collaboration on novelty of research. Here, five levels of collaboration are considered for each article–country, three levels of regions, and institutions, and novelty is measured with keywords information. Second, we investigate both the effect and moderating effect of research funding on novelty. Our findings show that there is a negative and significant relationship between scientific collaboration and novelty. Furthermore, funded papers show lower novelty than the unfunded, but funding does have a significant moderating effect on the relationship between collaboration and novelty. This study contributes by linking diverse levels of collaboration and funding sources to article’s novelty and thus extending the scope of bibliometric research of publications.

## Introduction

Scholars have long emphasized the role of scientific research in technological innovation and economic growth. Scientific knowledge provides background for fundamental understanding and subsequent practical applications for technological advancements [1–3]. In other words, scientific knowledge landscapes a foundation for ultimate technological and economic growth. In fact, a number of patents cite non-patent literature such as scientific publications as their prior art, and the number has grown dramatically over time [4]. Further, research with higher quality are more likely to be cited by patents, and, bilaterally, patents that cite scientific papers are likely to have higher impact and commercial value [5]. In this respect, accomplishing high-quality research outputs, mostly resulting in publications, must be the foremost goal in scholarly activities.

As one of common efforts to attempt high-quality scientific research, the number of international collaborations in science has increased substantially over the last three decades, and collaborative team science has become a universal trend [6–9]. Especially, the need of creative and interdisciplinary ideas to solve complex problems has driven the growth [10, 11]. In fact, previous literatures have shown that co-authored research papers tend to have higher impact when measured by the number of citations a paper gets [8, 12–14]. Yet, it is only recently that researchers have started to pay attention to city-level or institution-level scientific collaborations due to the availability of collecting and processing big publication data [15].

In addition to scientific collaborations, governments and organizations are well aware of the value of investments in science. Numerous initiatives and policies of funding programs have been established by public and private funding agencies to improve quality of research activities by enhancing research competitiveness, supporting interdisciplinary collaborations, and intensifying researcher networks to motivate knowledge exchange and creation [16–19]. Performance of funding has often been associated with the evaluation of efficiency of research systems, which is usually assessed by comparing spending on research and development (R&D) and publication impact [12, 20, 21]. However, the role of funding in bibliometrics remains largely unanswered since only few attempts were made to explore the effects of funding on published articles.

So far, when assessing scientific achievements of research papers, majority of work have utilized citation impact, since it implies the realization of peer-recognition of a paper and impact on science community [22, 23]. Citations, however, are regarded as a measure of impact, but not of research quality [21]. Besides impact, novelty or creativity is also considered valuable in scholarly outputs [11, 24], because ground-breaking ideas and opportunities are the ones to disrupt science and technology [9]. It should be noted that impact and novelty should be distinguished, and it is not always that novel papers get high citations [10, 25]. However, little is known about novelty of publications because of its difficulty to define and measure [26].

In this study, we focus on publication novelty as a scientific advancement with regard to collaboration and funding. First, we investigate the relationship between collaborations and novelty in publications. Novelty in scientific publications often incorporates new ideas and opportunities from interdisciplinarity across heterogenous research groups to solve practical problems [27–29]. Here, since interdisciplinarity, which results from scientific collaboration, is believed to bring out new research outputs, it is reasonable to expect some correlations between the collaboration and novelty. To the best of our knowledge, however, only few studies have examined the relationship between scientific collaborations and novelty. Here, we examine collaborations at country, three Nomenclature of Territorial Units for Statistics (NUTS) level regions, and institution level. We also measure novelty of articles with keywords information. Then, we aim to evaluate the role of funding in research novelty. To do so, we include a funding dummy variable as a moderator. The moderating effect is further examined with grouping our data by the number and quality of funding agencies. In short, this article aims to add to a line of bibliometric research of publications not only by examining different levels of collaboration but also by linking article’s novelty back to funding sources. Publication data is collected from Web of Science (WoS) Core Collection, and we limit our analysis on journal articles and proceedings papers in major scientific fields, Life Sciences & Biomedicine, Physical Sciences, and Technology, from European regions for the period of 2008–2017.

## The collaboration, funding, and novelty nexus

### Novelty in scholarly publications

Along with the access to publication data, substantial bibliometric research focusing on the impact of research has been conducted [22]. Systematic analysis of citations serves as a good proxy for scientific performance, since it implies the realization of peer-recognition of a paper and impact on science community [23]. Thus, majority of work has assessed the scientific achievement of research papers as: highly cited papers are the outcomes of high qualified research. Citations, however, are regarded as a measure of impact, not of research quality [21].

As an alternative to citation impact, novelty or creativity is also valued in scholarly activities, because ground-breaking ideas and opportunities are the ones to disrupt science and technology. In other words, scientific advancements are led by rare but important scientific discoveries which involve different perspectives on defining problems and combining diverse methods and models. Since research is a process of solving problems that involves diverse combinations of components such as problem defining, methods, etc., there can always be new combinations of those preexisting components. This often encourages further research and brings new creation of knowledge that can be described as novelty in publications [9, 11, 24, 26]. Novelty and citation impact of a paper should be distinguished because, first, it is not always that novel papers get high citations. Instead, higher variance in citations is observed among novel papers. While they have a higher opportunity for obtaining popularity, they also confront a higher uncertainty in impact. Also, novel studies may take longer time to gain recognition, which may undervalue the studies when relying on the measurement by short term citation windows [24]. In terms of the relationship between novelty and citation impact, studies have shown diverged results: positive [11, 30] or inverted U-shape [26]. Furthermore, according to *Lee*, *Walsh*, *and Wang* [10], the two measures depict different patterns in terms of collaborative works. For instance, the study claimed that there is an inverted U-shape relation between team size and novelty, while there is a continuous increasing relation between team size and impact. In addition, novelty is likely to be driven by knowledge variety, while impact is affected by team size.

Novelty in scientific publications is involved with new ideas and contributions that often come across disciplines [23]. In other words, exchanging and sharing knowledge from heterogenous research groups to solve practical problems is expected to increase novelty [27–29]. Specifically, scientific breakthroughs or inventions are achieved by new recombination of knowledge by extending the variety of knowledge pool and linking distant sources of knowledge. It is likely that novel outcomes are typically the result of recombining elements from more distant knowledge domains than within similar knowledge domains [2, 31]. These recombinant processes are regarded as the main mechanism for being creative or novel, which is demonstrated in publishing breakthrough-class papers [32].

Building on the idea of recombination of knowledge, scholars have attempted to describe or measure the novelty of publications by paper’s unusual combinations of references or keywords. First of all, *Uzzi et al*. [11] introduced the measurement of novelty by drawing on unusual or unexpected pairwise combinations of journals from references. This “atypical” combinations represent relatively new knowledge because they are rare in the combination domain. Given the bibliography data from WoS, frequencies of each journal pair were recorded to figure out whether it is atypical or conventional by comparing the observed frequency to the distribution of journal pairs that would have occurred by chance. This method was adopted by other studies [10, 23] to measure novelty of a paper. Moreover, *Wang*, *Veugelers*, *and Stephan* [24] defined a cosine similarity index to measure the ease of journal combinations that never have been made in the preceding three years. In short, atypical combination of references or the appearance of new reference combinations based on bibliography data was utilized to capture novelty.

However, some critics pointed out the use of references for measuring novelty, because most of the cited references are selected to contextualize the issues rather than to solve practical problems [29], and there are some risks that it might miss or overestimate the novelty depending on the use of references [28]. Plus, *Bornmann et al*. [32] tested whether the measurement of novelty by reference data converges to the assessment of novelty with F1000Prime data–a post publication peer review–and failed to show the validity.

Subsequently, new methods of measuring novelty based on keywords of a research paper emerged as an alternative: novelty measured by keyword combinations. *Bornmann et al*. [32] introduced the “Score K” defined as “the proportion of new keywords whereby newness is judged against the available keywords in one subject category from the same publication year” (p.5). In addition, *Carayol*, *Agenor*, *and Oscar* [30] applied the methodology of *Lee*, *Walsh*, *and Wang* [10] with the replacement of pairwise journal reference combinations with the pairwise keyword combinations. Lastly, *Yan*, *Tian*, *and Zhang* [26] used both of the two dimensions: new pairings of keywords in related research area [30] and the appearance of new keywords [32]. The study concluded that both measurements–new pairings and new appearance–capture similar phenomena.

### Scientific collaboration and its impact

According to *Cugmas*, *Mali*, *and Žiberna* [33], interactions among scientists and their scientific collaborations are important in the process of knowledge sharing and developing new ideas which are the prerequisites for scientific innovation. By pooling diverse perspectives through collaborations, it can contribute to the richness of using terminologies, research approaches, and methodologies in a science. Scientific collaborations can be defined and categorized in various ways depending on the units of actors, individual or organizational, and the type of information entailed in collaborating processes. Generally, scientific collaboration is operationalized through co-authorships in publications. While there may be several definitions and classifications of scientific collaborations, two components are common to all of them: a pursuit of a common goal and sharing knowledge.

Due to growing specialization in science labor, decrease in communication and travel costs, and the need to access interdisciplinary ideas and various database [10, 11], the number of internationally co-authored papers has grown significantly over the last three decades [8, 15]. Internationally co-authored papers take up to about 25% of WoS articles [7], and the increase in published outputs from Western Europe and United States is primarily driven by the growth of international collaborations [6]. Further, international collaboration between elite research teams became one of research trends, and the team size is getting bigger [9]. On one hand, there are still many countries in which domestic collaborations grow faster than international collaborations. In these countries, it is the interurban collaborations within a same country that reinforce research outputs [34]. Consequently, some studies tried to take account of intercity co-authorships both within and between countries [35–38]. However, most of the studies that deal with city level collaborations focused only on large or top publishing cities, and still, it needs more investigation since collaborations at city and institutional level have been analyzed not much due to data collecting and processing availability [15].

A great deal of studies has analyzed the effect of collaborations on scientific impact. Those studies have found that internationally co-authored research papers are likely to have higher impact in terms of citation counts [12–14, 21]. For example, *Narin*, *Stevens*, *and Whitlow* [39] found that papers with international co-authorship gained citations twice the rate of those from a single country, and *Wagner*, *Whetsell*, *and Leydesdorff* [8] and *Glänzel and Schubert* [40] also confirmed the positive relationship between co-authorship and impact in science disciplines and in all research disciplines, respectively. *Wagner et al*. [21] showed strong evidence of positive effect of openness, which represents percentage of internationally co-authored articles of a country and mobility of researchers, on citation impact using Scopus data and claimed that there are national benefits from participating in international scientific cooperation.

Similar results are found at city and institutional level studies. For instance, *Csomós*, *Vida*, *and Lengyel* [15] showed a positive correlation between a Jaccard index to measure relative strength of intercity collaborations and a binary variable of highly cited papers. Moreover, *Abbasi and Jaafari* [41] explored the correlation between institutional collaboration types and their impact on research. The types were classified into intra- or inter-departmental collaborations and intra- or inter-institutional collaborations. While all types of collaborations had positive and significant effect on impact, the results represented that inter- departmental and institutional collaborations show higher correlation with average number of citations than intra- collaborations. However, publications with co-authoring from different departments and institutions inside the same country had higher impact than those across countries.

In the meantime, collaborations across heterogeneous groups foster knowledge dissemination within a wider range of information, thus increasing the possibility to bring out new research outputs [27–29]. As interdisciplinarity which results from collaboration is believed to enhance novelty, it is reasonable to expect some correlations between the two elements. However, only few trials have been made to evaluate the collaboration effect on novelty of publications. *Wagner*, *Whetsell*, *and Mukherjee* [23] analyzed the relationship between the number of countries per article and novelty and found that international collaboration indicates low novelty, and *Lee*, *Walsh*, *and Wang* [10] showed an inverted-U shape relationship between team size and novelty. Additionally, *Wu*, *Wang*, *and Evans* [9] revealed that smaller teams tend to create disruptive research by exploring novel ideas from less-popular works, while big teams tend to rely on recent successes and ongoing stream of funding. The findings from these studies on collaboration and novelty are contrary to the results from other studies that show citation impact rises with team size.

### The role of funding in publications

Numerous policies and initiatives of funding programs have been established by national governments, organizations, and private agencies in order to enhance competitiveness and facilitate interdisciplinary coordination of research activities [19, 41, 42]. As an example, a pan-European funding program, the Framework Programs, by the European Commission is designed to pool researchers and resources so that all countries and regions in the EU can participate in international collaborations and move forward to frontier knowledge [18]. The research funding is recognized to motivate new exchange of knowledge and intensify contact networks among researchers, constituting instruments for deepening collaborations and knowledge creation, where scientific and technological capabilities are often concentrated in typical geographic boundaries [16, 17].

Measuring the performance of funding has often been associated with the evaluation of efficiency and effectiveness of a country’s research system which is assessed by spending on R&D and publication impact. However, results of analyses on the relationship between R&D funding and nation’s citation impact are not consistent. While positive relationship is observed in some studies [19, 21], others show a negative or only a slight effect of funding [12, 20]. In addition, few attempts have been made to assess the effect of funding on individual research papers since publications are the primary output of academic activities, but they also show inconclusive results. While *Li*, *Azoulay*, *and Sampat* [5] represents positive correlations between grants award from National Institutes of Health (NIH) and possibility of being cited by commercial patents, *Yan*, *Tian*, *and Zhang* [26] depicts insignificant effect of funding on an article’s citation impact. Yet little is known about whether and in what direction the acquisition of funding has impact on scientific output [42].

Moreover, as seen above, measuring the effect of funding is often coupled with research impact. Several scholars, however, are concerned about this impact agenda in research funding [43]. Research with less potential, which is likely to be new, would be alienated in research funding decisions or under-funded because of the impact agenda, while research with potential impact would be prioritized, even if they are equally valuable. In other words, the so-called ‘blue-skies’ research may not be competitive in funding environment. Placing greater value on the popular research topics with practical relevance at the expense of others might narrow the range of research and ultimately challenge new discoveries that have no obvious application at the time. In terms of novelty of research, however, the role of funding has been mostly unanswered.

## Materials and methods

### Data

**Fig 1:**
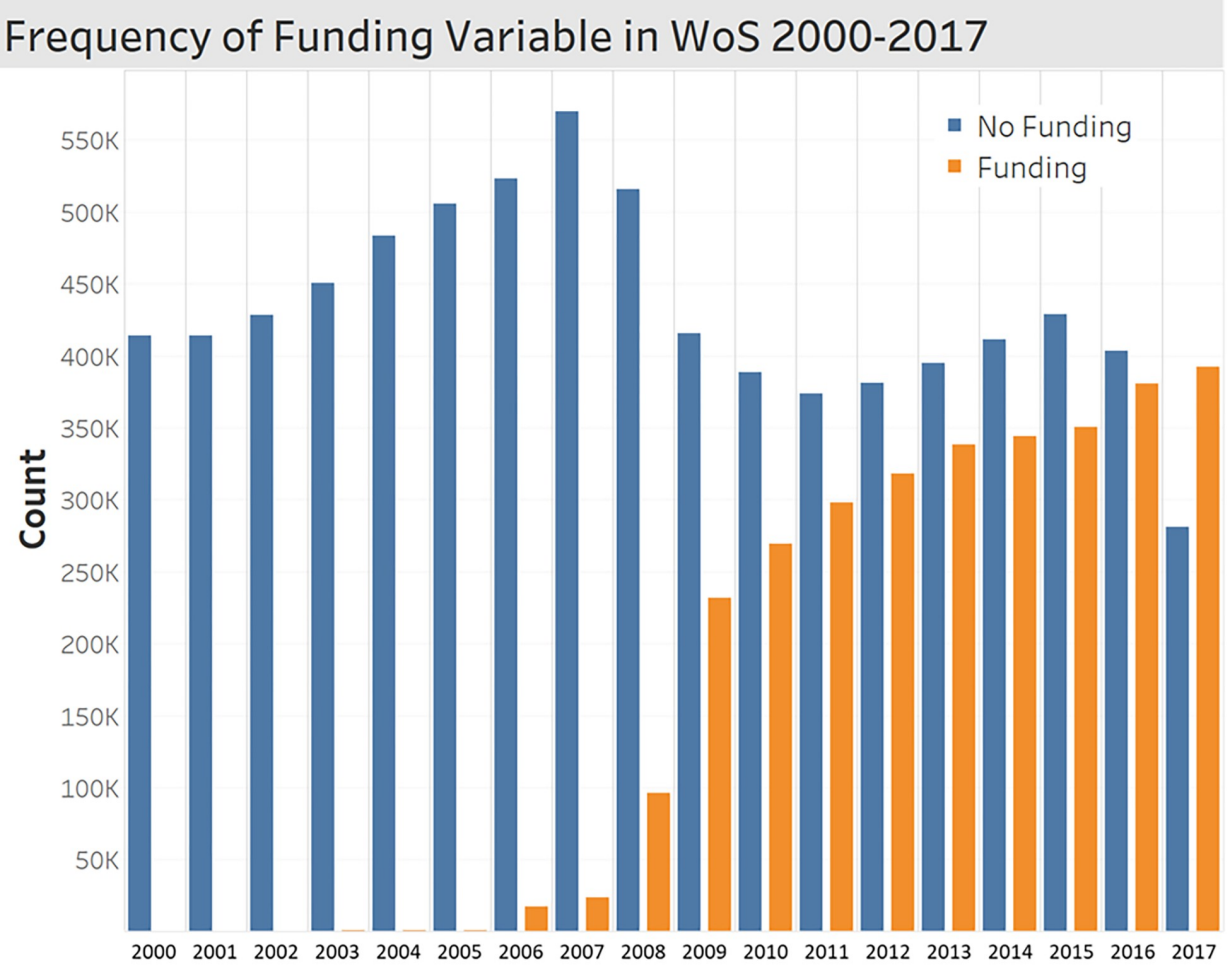
Frequency of funding variable in WoS 2000–2017.

We retrieved publication data from the WoS database. It is the most representative and widely used database for bibliometric analyses and covers a wide range of information on published research [22]. For our analysis, first, we limited our sample to journal articles and proceedings papers in major scientific fields–Life Sciences & Biomedicine, Physical Sciences, and Technology. Second, we extracted papers that were written by authors with affiliation located in European regions. To do so, upon the affiliation address information provided, we obtained the NUTS codes by additional geo-coding process and filtered publications from European regions accordingly. Moreover, time periods were restricted to 2008–2017 to cope with the balance of variable observations. From the WoS dataset, we noticed that funding information starts to increase dramatically from 2008, while the frequencies of most of our variables show consistency over time. As shown in Fig 1, only few papers have funding details until 2007. This should be because of changes in requirement for researchers to report funding information in acknowledgements. Regardless of any reason, our sample includes data from 2008 to maintain the consistency of frequencies of observations. Lastly, we excluded research papers with zero backward citations and any missing values. The final dataset contains 3,077,225 observations.

### Methods

#### Measurement of novelty

In this study, we focus on novelty of publications. Here, novelty is defined as the unprecedented pop-up knowledge which may not be connected to innovativeness at the moment but still has potential for groundbreaking outcomes. In this sense, our study adopts *Bornmann et al*. [32]’s keyword approach–capturing new keyword combinations–to measure novelty of scientific papers. Based on the keyword information provided by the WoS, first, text pre-processing has been conducted. This step is needed to avoid the case of recognizing the same-meaning keywords as different keywords. Text pre-processing includes converting all the letters to the lower letters, removing the symbols, and lemmatization. By lemmatizing the keywords, the base or dictionary form of a word replaces the similar-meaning keywords. Then, we divide keywords into novel and non-novel keywords. Novel keywords are the new keywords that have never appeared in the past five years, and non-novel keywords are the ones which have already appeared in the past five years. To do so, we compare the list of keywords from scientific papers published in year *t* and keywords from scientific papers published between year *t-1* and *t-5*. Once the list of novel and non-novel keywords is obtained, we count the number of novel and non-novel keywords for each paper and measure the novelty as a proportion of new keywords to keywords in total. A paper *i*’s novelty is calculated as follows:

$${Novelty}_{i}=\frac{k_{in}}{k_{i}}$$ (1)

where *k*_*in*_ is the number of new keywords in a paper *i*, and *k*_*i*_ is the total number of keywords in paper *i*.

Lastly, we take natural logarithm transformation of novelty measure as a dependent variable to alleviate high-skewness and roughly normalize the distribution of the variable.

#### Independent variables

The first aim of this study is to figure out the effect of scientific collaborations on novelty. In our study, geographical diversity is considered in collaboration which is supposed to be beneficial by extending knowledge pool but at the same time is influenced largely by coordination costs. Through collaboration, researchers from diverse geographical background are endowed access to sophisticated and specialized knowledge from various local sources. On the other hand, research collaborations with large heterogeneity are difficult to manage. Further, geographical proximity is considered a salient factor to draw collective fine outcomes [44]. To analyze whether geographically diverse collaborations increase research novelty, this paper measures research collaborations as the count of different countries–following *Wagner*, *Whetsell*, *and Mukherjee* [23] ‘s work–and NUTS1-3 regions. Further, according to *Larivière et al*. [45], international collaborations and interinstitutional collaborations may not have same gains. Thus, we also measure the count of different institutions per article from the affiliation information. In short, we measure all macro-meso-micro level of geographical collaborations: for each paper *i*, each number of five collaboration level is calculated. For example, a paper with two authors from Gaziantep University, Turkey and Assistance Publique—Hôpitaux de Paris, France has two counts for all levels of collaboration.

Secondly, to analyze whether funding works as a tool to enhance research novelty, we identified papers’ funding information and created a dummy variable: if a paper *i* was assigned at least one grant, then the dummy indicates 1; otherwise, 0. First, we investigate whether funded works or non-funded ‘blue-skies’ works have higher novelty. Then, to assess the underlying mechanism of funding, we also include funding as a moderator between collaboration and novelty to see if the funding affects the direction and/or strength of the relationship between the two variables. Furthermore, we conduct additional analyses with more detailed categorical variables of funding–number of funding agencies and class of funding agencies–to compare their effects.

#### Control variables

According to previous studies, many factors such as team size or field diversity have been shown to influence publication outputs including citation impact and novelty. Following the studies, we control for the number of authors [23, 24, 26, 28, 41], subjects [23, 26], and references [10, 23, 24, 28]. We also add a country border dummy to see if the effects of collaborations between regions or institutions are different when they are within the same country or not.

#### Estimation models

Given that the dependent variable, log-transformed novelty, is a continuous variable, we adopt ordinary least squares (OLS) regression models. Regressions are conducted five times for each level of collaboration independent variables. We also add funding variables and interaction terms of collaboration and funding to analyze the moderating effects. Moderating effects can be confirmed through the significance of interaction terms. All models control for the number of authors, subjects, and references, and a country border. We estimate the model with robust standard errors to reduce estimation errors from heterogeneity. Also, the independent variables, five levels of collaboration, are mean centered to lessen the correlation problem that might occur when including interaction terms and to make interpretation easier. The regression model used in this study is as follows:

$${lnNovelty}_{i}=\beta_{0}+{\beta_{1}Collaboration}_{i}+{\beta_{2}Funding}_{i}+{\beta_{3}(Collaboration\times Funding)}_{i}+{\beta_{4-8}Controls}_{i}+e_{i}$$ (2)

where *i* stands for each publication.

Besides, some of the prior studies used field fixed effects since novelty can vary significantly across disciplines. Those studies, however, include non-scientific research fields such as Social Sciences or Arts & Humanities [10, 11, 23, 30]. On the other hand, this paper assumes that there would be little disciplinary effects between the three fields we use since *Wagner*, *Whetsell*, *and Mukherjee* [23] categorized these fields into a “Science” field among Social Sciences and Arts & Humanities. Additional estimation models for different fields will be run as robustness checks.

## Results

### Descriptive analysis

**Fig 2:**
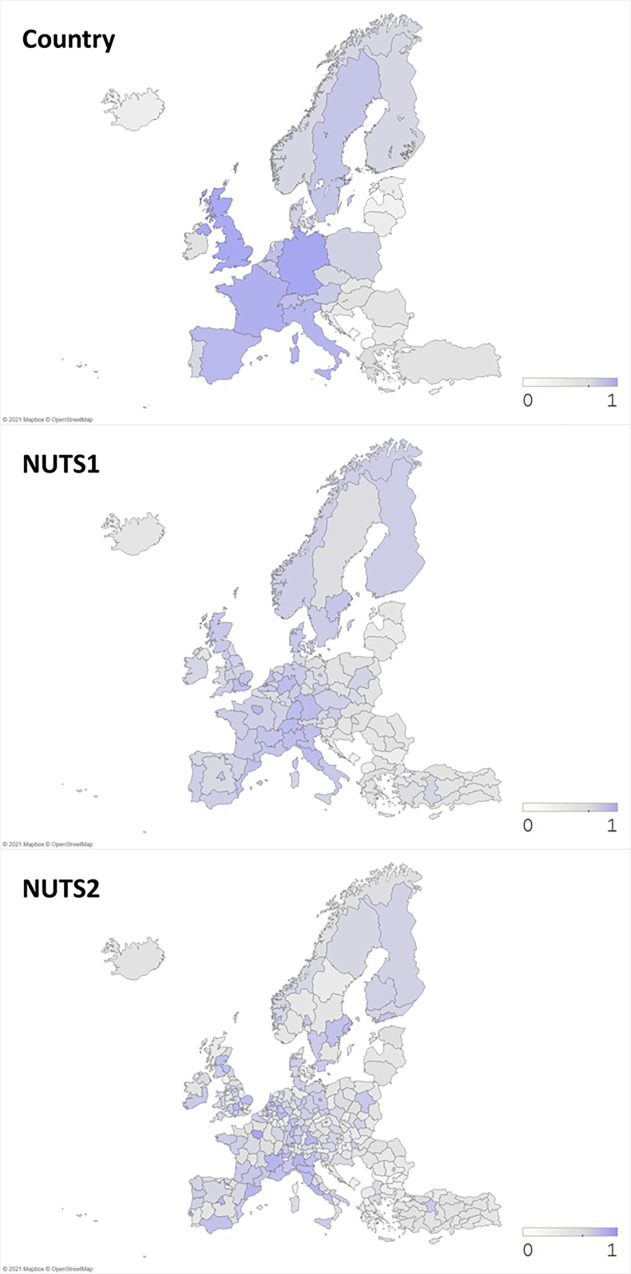
Number of scientific collaborations (in normalized log scale). Source: Authors’ illustration.

**Fig 3:**
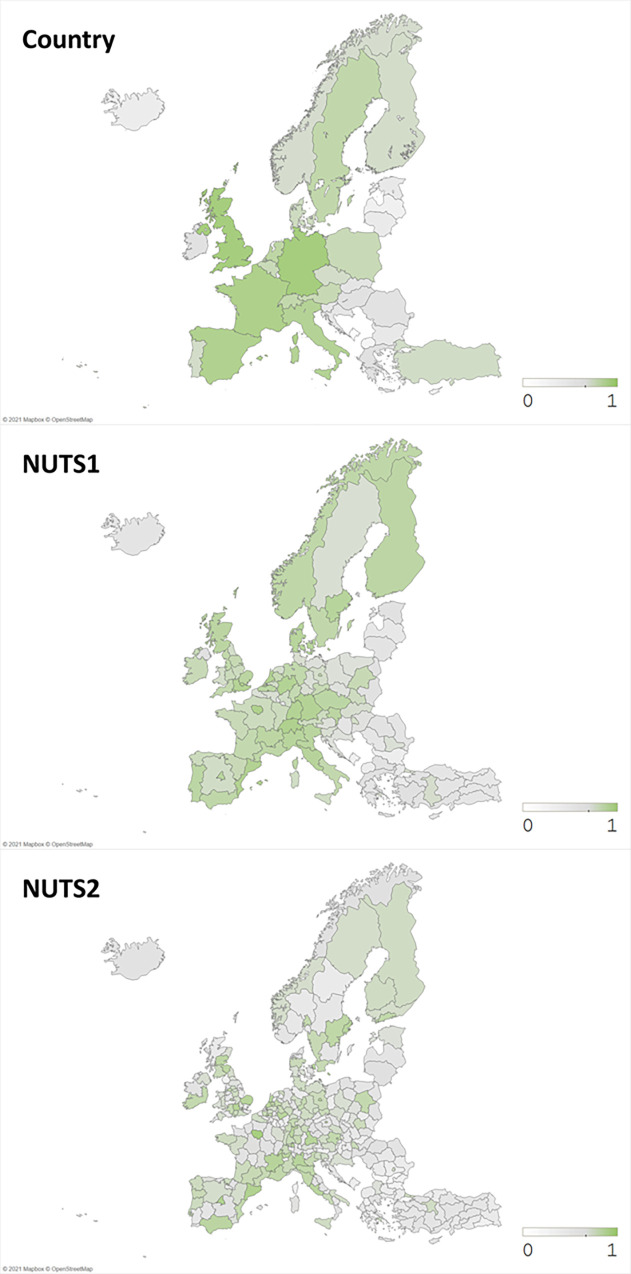
Number of funded publications (in normalized log scale). Source: Authors’ illustration.

**Fig 4:**
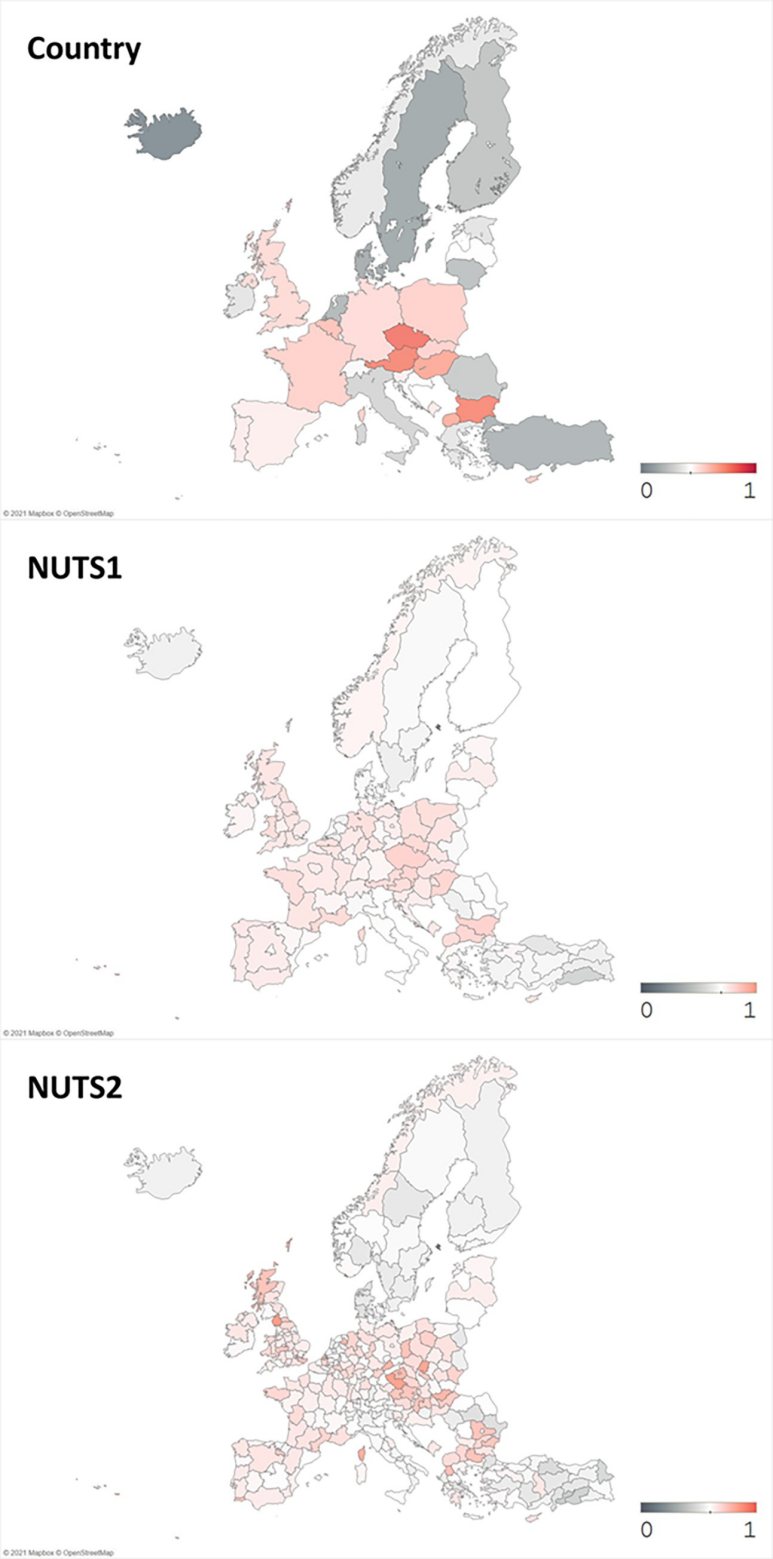
Average novelty of publications (in normalized log scale). Source: Authors’ illustration.

In advance to estimating our model, we first check our dataset’s reliability by exploring geographical distributions of our focal variables. Figs 2–4 illustrate our data on variables of scientific collaboration, research funding, and publication novelty in Life Science & Biomedicine, Physical Sciences, and Technology field from 2008 to 2017 in Europe. First of all, Fig 2 depicts inter- national and regional co-authorships between European regions. As the color gets darker, it means that the number of scientific collaborations is higher than the ones in lighter color. It is evident that the collaborative activities are concentrated in Western Europe countries, as already shown in previous studies, e.g., *Adams* [6], especially in Germany, United Kingdom, France, Italy, and Spain. At regional level, western and northern regions rank in top co-publishers. Lists of top co-publishing countries and NUTS2 regions are shown in S1 Table. Next, Fig 3 demonstrates the number of funded publications for each country and region, which is in a similar pattern to Fig 2.

On the other hand, Fig 4 shows the average novelty of publications, which displays a quite different pattern from Figs 2 and 3. As the color gets closer to grey, it represents lower novelty and as the color gets closer to red, it means higher novelty. We observe some spots of high publication novelty in regions from Eastern Europe along with western regions. While top regions in the number of co-publications and funded publications shown in Figs 2 and 3 correspond to top publishing regions known as Western and Northeastern Europe [16], some regions with high average novelty shown in Fig 4 do not match with those well-known regions. In other words, we realize that large number of publications nor the collaborations does not stand for high research novelty.

In this study, we have measured the novelty by the first appearance of new keywords, thus possible explanations to this figure are: first, institutions in peripheral regions are likely to come up with some novel keywords, while big and popular institutions in well-known regions would stick to their specific research era and keywords. *Wu*, *Wang*, *and Evans* [9] has found that large teams focus on popular and high impact works, while small teams build on less popular ideas which may appear as novel keywords. Thinking that core regions are likely to participate in big research teams, peripheral regions in small collaborative teams are the ones who work on novel ideas. Furthermore, novel ideas have high risks that those can either result in influential outcomes or just fade away [30]. Only the successful works among them later become the source of big-team’s high impact research. Thus, it is reasonable for collaboration, funding, and novelty variables to vary in their patterns when plotted on geographical maps.

**Fig 5:**
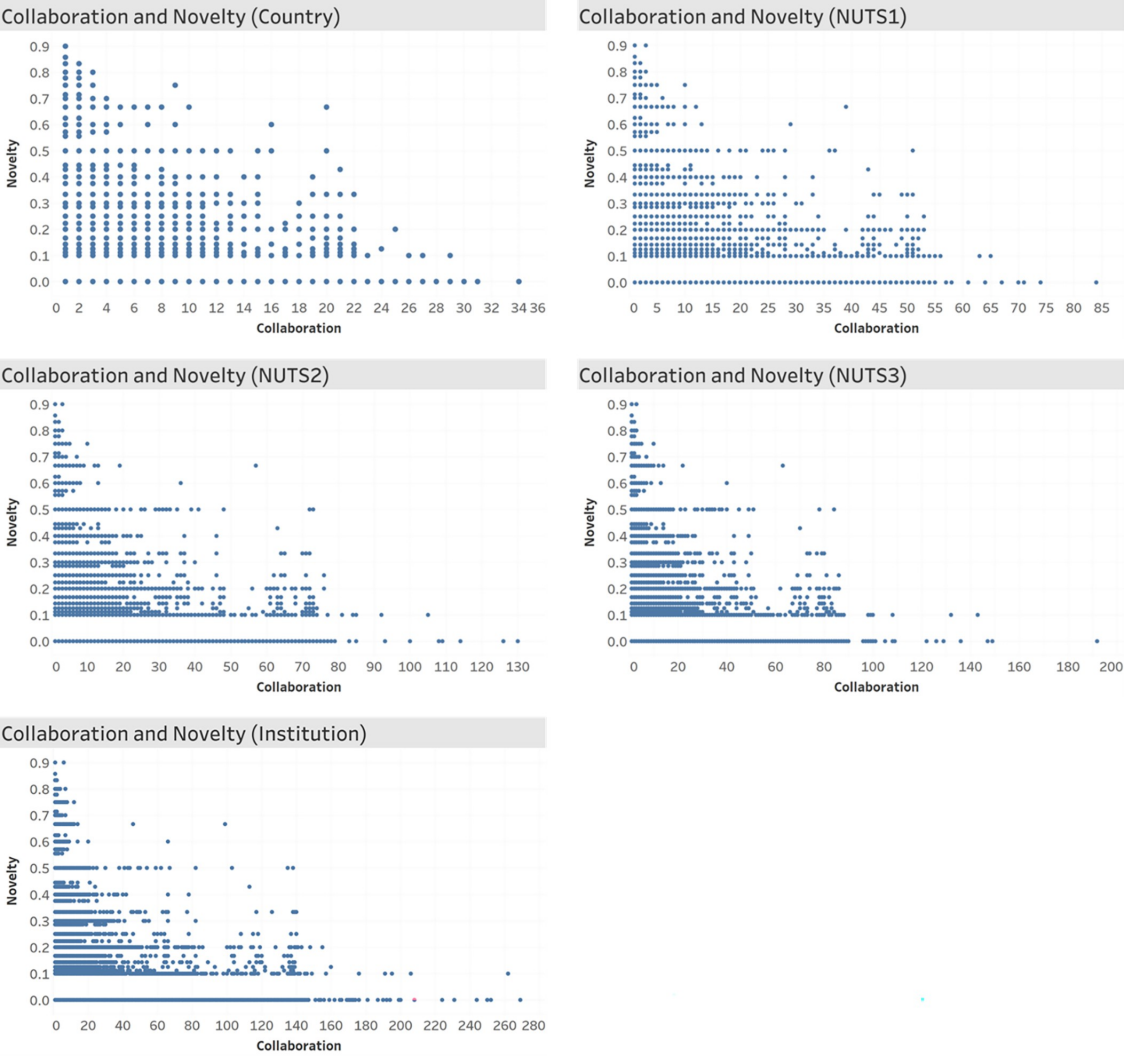
Scatterplots for collaboration and novelty.

As we have checked the reliability of our data and variables through Figs 2–4, we now present descriptive results for individual paper level analysis. Fig 5 represents the scatterplots of collaborations and novelty. Since negative relationship between the two variables is observed at every collaboration type, we can expect that collaborations have negative effect on publication’s novelty.

Table 1 depicts summary statistics of the variables and their correlation matrix. First, means of collaboration count gradually increases from country (*Country*) to region (*NUTS1*, *NUTS2*, and *NUTS3*) and to organization (*Institution*) level– 1.3, 1.59, 1.71, 1.8, and 2.57, respectively. In addition, the mean of a funding dummy (*Fund*.*d*)– 0.64 –tells us that about 64% of research papers from European regions are funded. In the correlation matrix, we can observe that collaboration counts for each collaboration type are negatively correlated with novelty (*Novelty*).

**Table 1:** Summary statistics.

Summary statistics							Correlation matrix									
	Variable	Obs.	Mean	Std. Dev.	Min	Max	1	2	3	4	5	6	7	8	9	10
**1**	*Novelty*	3,077,225	.0369	.0771	0	.9										
**2**	*Country*	3,077,225	1.2979	.8363	1	34	-.0060									
**3**	*NUTS1*	3,077,225	1.5892	1.4597	1	84	-.0125	.8524								
**4**	*NUTS2*	3,077,225	1.709	1.8364	1	126	-.0150	.8044	.9699							
**5**	*NUTS3*	3,077,225	1.799	2.0377	1	149	-.0167	.7735	.9484	.9857						
**6**	*Institution*	3,077,225	2.5689	3.2632	1	413	-.0324	.6733	.8500	.8998	.9810					
**7**	*Fund*.*d*	3,077,225	.6353	.4813	0	1	.0009	.1023	.0778	.0723	.0680	.0615				
**8**	*#Author*	3,077,225	6.7243	43.8041	1	5052	-.0091	.4639	.6184	.6916	.6992	.7126	.0280			
**9**	*#Sub*	3,077,225	1.6786	.8764	1	6	-.0057	-.0204	-.0272	-.0264	-.0267	-.0324	.0170	-.0079		
**10**	*#Ref*	3,077,225	3.0996	2.6098	1	524	.0633	.0216	.0170	.0119	.0096	.0022	.0356	-.0040	-.0171	
**11**	*Cnt*.*d*	3,077,225	.7941	.4044	0	1	.0036	-.6996	-.4771	-.4078	-.3781	-.2842	-.1254	-.0689	.0079	-.0280

All correlations are significant at p < .05 except for ρ*(Fund*.*d*, *Novelty)*.

### Scientific collaboration, research funding, and novelty in publications

Given the importance of collaboration effect in science, our first research question is how novelty in scientific publication responds to the collaboration. Table 2 estimates the effects of different levels of collaborations on novelty. All of five models show that collaboration has a negative and significant effect on novelty, which means collaborative works tend to have lower novelty than the works from within a same country, region, or institution. Furthermore, the coefficients of country dummy in models (2)—(5) show positive signs, which means that inter- city and institutional collaborations within a single country have higher novelty than those across several countries. From these results, we can assume that novelty decays with geographic distances involved in scientific collaborations.

**Table 2:** Regression results 1.

	Dependent variable: Novelty				
	(1)	(2)	(3)	(4)	(5)
**Intercept**	-1.8866*** (.0016)	-1.9006*** (.0019)	-1.9005*** (.0019)	-1.9006*** (.0019)	-1.8985*** (.0019)
**Country** ^a^	-.019*** (.0014)				
**NUTS1** ^a^		-.0162*** (.001)			
**NUTS2** ^a^			-.0161*** (.0009)		
**NUTS3** ^a^				-.0156*** (.0008)	
**Institution** ^a^					-.0176*** (.0012)
**Funding dummy**	-.091*** (.0011)	-.0896*** (.0011)	-.0894*** (.0011)	-.0893*** (.0011)	-.088*** (.0011)
**Col⊆Funding dummy**	.0078*** (.0016)	.0092*** (.001)	.0086*** (.0009)	.0081*** (.0009)	.0071*** (.0012)
**# of authors**	-.0001+(.0000)	-.0000 (.0000)	.0001*** (.0000)	.0002*** (.0000)	.0006*** (.0000)
**# of subjects**	-.0011* (.0006)	-.0013* (.0006)	-.0014* (.0006)	-.0014* (.0006)	-.0019* (.0006)
**# of references**	-.015*** (.0002)	-.015*** (.0002)	-.015*** (.0002)	-.0146*** (.0002)	-.015*** (.0002)
**Country dummy**		.0157*** (.0014)	.0147*** (.0013)	.0145*** (.0013)	.0072*** (.0014)
**N**	754,285	754,285	754,285	754,285	754,285
**Prob>F**	.0000	.0000	.0000	.0000	.0000
**Vif**	2.31	2.34	2.40	2.38	2.42
**R** ^**2**^	.0228	.0237	.0239	.0241	.0270

+p < .1

*p < .05

**p < .01

***p < .001.

^a^ Collaboration variable.

**Fig 6:**
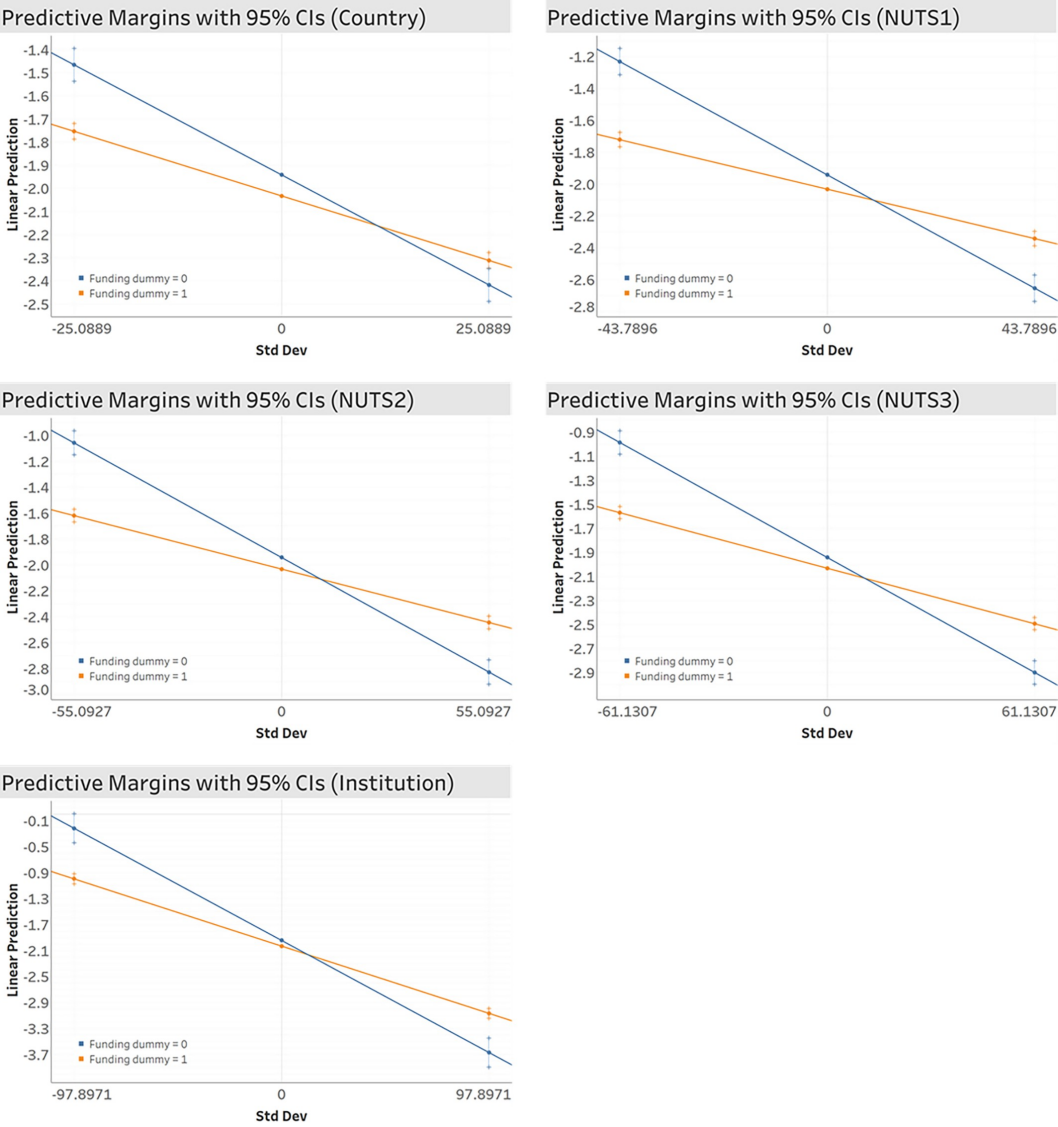
Margins plots for funding dummy and novelty. Std Dev: Standard Deviation of collaboration variable.

Also, the results in Table 2 clearly show that non-funded ‘blue-skies’ works have higher novelty than funded works. However, to further assess the role of funding, we added a funding variable as a moderator to see whether the funding changes the nature of the relationship between collaboration and novelty. The moderating effect is measured by the interaction term of collaboration and funding dummy. According to the coefficients of interaction term *Col⊆Funding* in Table 2, we verify that funding has a significant positive moderating effect: it alleviates the negative effect of collaboration on novelty. In other words, while remote collaborations have negative effect on novelty, however, among the collaborative works, funded works are more novel than the unfunded ones. This phenomenon is captured in margins plots shown in Fig 6. The funded works (in orange line, funding dummy = 1) catch up the unfunded works (in blue line, funding dummy = 0) at one point and show higher novelty.

In addition, we attempted to identify details of funding effect by additional analyses with categorical variables of the number of funding agencies and class of funding agencies. First, in terms of the number of funding agencies, we divide publications by whether the paper was funded by multiple agencies, single agency, or no agency (no funding) and see if there are differences in the effects. When setting no agency as a base-level, the results from Table 3 show that papers funded by multiple agencies have the lowest novelty. Nevertheless, both interaction variables are positive and significant throughout all levels of collaboration except for *Col⊆Single* at institutional level. Also, *Col⊆Multiple* coefficients are higher than *Col⊆Single*, showing that moderating effect is ranked in multiple, single, and no agency order.

**Table 3:** Regression results 2.

	Dependent variable: Novelty				
	(1)	(2)	(3)	(4)	(5)
**Intercept**	-1.8865*** (.0016)	-1.8978*** (.0019)	-1.8977*** (.0019)	-1.8978*** (.0019)	-1.8957*** (.0019)
**Country** ^a^	-.0189*** (.0014)				
**NUTS1** ^a^		-.0167*** (.001)			
**NUTS2** ^a^			-.0163*** (.0009)		
**NUTS3** ^a^				-.0158*** (.0008)	
**Institution** ^a^					-.0176*** (.0012)
**Multiple**	-.1034*** (.0011)	-.1018*** (.0011)	-.1016*** (.0011)	-.1015*** (.0011)	-.0997*** (.0012)
**Single**	-.0684*** (.0014)	-.068*** (0014)	-.0679*** (0014)	-.068*** (0014)	-.0681*** (.0014)
**Col⊆Multiple**	.0111*** (.0016)	.0119*** (.0011)	.011*** (.001)	.0104*** (.0009)	.0088*** (.0012)
**Col⊆Single**	.0048* (.002)	.0035** (.0013)	.0026* (.0012)	.0023* (.0011)	.0021 (.0014)
**# of authors**	-.0001*(.0000)	.0000 (.0000)	.0001* (.0000)	.0001*** (.0000)	.0005*** (.0000)
**# of subjects**	-.0014* (.0006)	-.0016** (.0006)	-.0016** (.0006)	-.0017** (.0006)	-.0022*** (.0006)
**# of references**	-.0145*** (.0002)	-.0145*** (.0002)	-.0145** (.0002)	-.0145*** (.0002)	-.0145*** (.0002)
**Country dummy**		.0125*** (.0014)	.0116*** (.0014)	.0114*** (.0013)	.0044** (.0014)
**N**	754,285	754,285	754,285	754,285	754,285
**Prob>F**	.0000	.0000	.0000	.0000	.0000
**Vif**	2.06	2.13	2.18	2.16	2.20
**R** ^**2**^	.0239	.0247	.0250	.0252	.0280

+p < .1

*p < .05

**p < .01

***p < .001.

^a^ Collaboration variable.

Next, to figure out whether the effects vary depending on the quality of funding agencies, we classified the agencies into high, mid, and low agency. To do so, we counted the frequency of funding agencies among the whole dataset and assumed that if typical agency appears many times, then the size of grant the agency is supporting would be large, which means that the agency is likely to be one of top tier funding agencies. Following, we specified top 5% and low 5% funding agencies and added a categorical variable of high, mid, low, and no agency accordingly. When setting no agency as a base-level, Table 4 again shows that funded works have lower novelty. Interaction variables *Col⊆High* and *Col⊆Mid* are positive and significant throughout all levels of collaboration except for *Col⊆Mid* at institutional level, while *Col⊆Low* variables are positive but not significant. Moreover, moderating effect is ranked in high, mid, low, and no agency order except at the country level.

**Table 4:** Regression results 3.

	Dependent variable: Novelty				
	(1)	(2)	(3)	(4)	(5)
**Intercept**	-1.8866*** (.0016)	-1.9*** (.0019)	-1.8999*** (.0019)	-1.9*** (.0019)	-1.8978*** (.0019)
**Country** ^a^	-.019*** (.0014)				
**NUTS1** ^a^		-.0163*** (.001)			
**NUTS2** ^a^			-.0161*** (.0009)		
**NUTS3** ^a^				-.0156*** (.0008)	
**Institution** ^a^					-.0176*** (.0012)
**High**	-.0936*** (.0011)	-.0922*** (.0011)	-.0919*** (.0011)	-.0919*** (.0011)	-.0906*** (.0011)
**Mid**	-.0807*** (.0024)	-.0799*** (.0024)	-.0796*** (.0024)	-.0795*** (.0024)	-.0784*** (.0025)
**Low**	-.076*** (.0021)	-.0749*** (.0021)	-.0747*** (.0021)	-.0745*** (.0021)	-.0732*** (.0021)
**Col⊆High**	.0083*** (.0016)	.0099*** (.001)	.0094*** (.001)	.0089*** (.0009)	.0078*** (.0012)
**Col⊆Mid**	.0102** (.0033)	.0051** (.0022)	.0043* (.0019)	.0035* (.0017)	.0017 (.0016)
**Col⊆Low**	.0048 (.0031)	.0033+ (.0019)	.0011 (.0017)	.0007 (.0015)	.0001 (.0015)
**# of authors**	-.0001+ (.0000)	.0000 (.0000)	.0001* (.0000)	.0001*** (.0000)	.0005*** (.0000)
**# of subjects**	-.0012* (.0006)	-.0014* (.0006)	-.0014* (.0006)	-.0015** (.0006)	-.002*** (.0006)
**# of references**	-.0146*** (.0002)	-.0145*** (.0002)	-.0146*** (.0002)	-.0146*** (.0002)	-.0146*** (.0002)
**Country dummy**		.015*** (.0014)	.014*** (.0013)	.0138*** (.0013)	.0065*** (.0014)
**N**	754,285	754,285	754,285	754,285	754,285
**Prob>F**	.0000	.0000	.0000	.0000	.0000
**Vif**	1.83	1.90	1.94	1.92	1.95
**R** ^**2**^	.0230	.0238	.0241	.0243	.0272

+p < .1

*p < .05

**p < .01

***p < .001.

^a^ Collaboration variable.

### Robustness tests

In this section, we discuss the robustness of our models in Table 2 by subdividing our samples by different research fields (called as ‘subheading’ in the WoS), since results may vary depending on the disciplines. By excluding the sample without subheading information (NA, 17.60%), the final sample for robustness check includes 2,535,507 publications with the disciplines Life Science & Biomedicine (61.73%), Physical Sciences (27.20%), and Technology (11.07%) (See Table 5).

**Table 5:** Subheading types.

Subheading	Freq.	Percent	Cum.
**Life Sciences & Biomedicine**	1,565,062	61.73	61.73
**Physical Sciences**	689,708	27.20	88.93
**Technology**	280,737	11.07	100.00
**Total**	2,535,507	100.00	

Not Available (NA) (17.60%) excluded.

We represent the results for each field at NUTS2 level in Table 6, and full results are appended in S2 Table. Although some of the coefficients show weaker significance, e.g., the moderating effect of funding in Technology, the overall results are generally consistent with the results in Table 2. In other words, collaborations have negative effects on novelty, and funding variables show positive moderating effects in all three disciplines, thus fulfilling the robustness.

**Table 6:** Robustness check.

	Dependent variable: Novelty		
	Life Science & Biomedicine	Physical Sciences	Technology
**Intercept**	-1.9323*** (.0036)	-1.8321*** (.0058)	-1.6353*** (.0125)
**NUTS2** ^a^	-.0054** (.0016)	-.0154*** (.0018)	-.0224*** (.005)
**Funding dummy**	-.0993*** (.0024)	-.1057*** (.0038)	-.118*** (.0087)
**Col⊆Funding dummy**	.0085*** (.0012)	.0067*** (.0018)	.0103+ (.0056)
**# of authors**	-.003*** (.0007)	.0003*** (.0000)	.0001 (.0002)
**# of subjects**	-.0103*** (.0008)	.0079*** (.002)	-.0066* (.0026)
**# of references**	-.0086*** (.0002)	-.0165*** (.0006)	-.0365*** (.0012)
**Country dummy**	.0126*** (.002)	.0305*** (.0028)	.0101 (.0069)
**N**	378,955	182,398	61,449
**Prob>F**	.0000	.0000	.0000
**Vif**	2.37	4.26	3.26
**R** ^**2**^	.0208	.0272	.0390

+p < .1

*p < .05

**p < .01

***p < .001.

^a^ Collaboration variable.

## Discussion and conclusion

As new ideas and contributions bring disruptive advancements in science and technology, we focused on the value of novelty in scientific publications. Revisiting the research questions, this study examined, first, the relationship between scientific collaboration and novelty of research, and second, the effect of research funding on novelty. To do so, we retrieved scientific publication data from the WoS that were published in major scientific fields from European regions between 2008–2017. Then, we counted collaborations per article at country, NUTS1-3 regions, and institution level and evaluated novelty using research paper keywords. Further, we generated funding variables based on the funding information of each paper to evaluate the role of funding. To figure out the relationship between collaboration and novelty, we conducted OLS regressions and confirmed that there is a negative and significant relationship between co-authorship and novelty. Furthermore, by adding funding variables and interaction terms of collaboration and funding, we could confirm that funded works showed lower novelty than non-funded works, however, funding does have a significant and positive moderating effect on the relationship between collaboration and novelty, especially, for papers funded by multiple and top agencies.

We could, first, confirm the negative effect of collaborations on novelty. A collaborative learning process is a kind of an agreement among several researchers and organizations through exchanging, modifying, or elaborating ideas [46]. This can enlarge readership, and thus, citation impact [13], but may result in filtering out some disruptive ideas or outliers when the collaboration size gets bigger. *Wu*, *Wang*, *and Evans* [9] has also argued that among high impact papers, it is likely for smaller teams to create disruptive outcomes and for larger teams to conduct developmental research. In other words, while smaller groups tend to explore and raise promising ideas from less popular findings, larger groups of researchers tend to rely on recent successes and refine or develop those based on common research designs. Furthermore, novelty may be restrained since collaborative works mostly rely on distant communications. Teams from diverse geographical areas have no choice but to use communication technologies to share and exchange knowledge. However, tacit knowledge and inherited know-hows which are critical components in scientific knowledge and innovations are not likely to be transmitted through those channels. Tacit knowledge is rather transferred through face-to-face communications or physically proximate distant, and it becomes even more immobile for higher complex knowledge [47]. Thus, creativity or innovative ideas decay among distances, which decreases the opportunity for novel outcomes [23]. Consequently, we can assume that there are distant decay effects in publication novelty when collaborating with remote countries or regions.

Secondly, we find that non-funded ‘blue-skies’ research have higher novelty than funded works. Especially, research recognized by multiple or top agencies were likely to have the lowest novelty, and this confirms the pervasive impact agenda in funding environment. However, we also find a positive moderating effect of funding, which indicates a positive function of funding as an instrument to motivate explorative research, i.e., the acquisition of funding mitigates the negative effect of distant collaborations on novelty. In other words, funding allows the collaborative researchers to overcome the difficulties or restrictions that come along with remote collaborations and to pursue novel outcomes. Especially, this positive effect is the highest when researchers are awarded by multiple funding agencies or top tier agencies. This highlights the importance of funding in science activities that it not only contributes to the scientific advancement but also enhances the opportunity of novel outcomes.

Our findings contribute, first, by examining macro-meso-micro levels of collaborations in Europe and by linking them to the value of novelty in scholarly activities. Also, we could reveal the role of funding in research novelty which has been mostly unanswered. Here, we utilized moderating variables of funding in our analyses to examine whether funding does leverage academic activities and outputs, and we further categorized the effects of funding by the number of funding agencies and the class of funding agencies, which could provide us details of funding’s effectiveness. This estimation method can be used in further studies to assess the effectiveness of research funding in science.

However, our research has some limitations that could be complemented in future research. Currently, our data is limited to 2008–2017, which should be extended in further studies. By expanding the time frame of observations to recent years, we can confirm recent trends in research activities. Moreover, it is highly likely that there have been significant challenges and responding changes in scholarly activities, e.g., collaborations and grant awarding, due to the breakout of COVID-19 pandemic. It would be very interesting to figure out some specific inclinations observed during those times compared to pre-pandemic periods. Our data is also limited to papers in scientific disciplines. As prior studies have shown, there may be different results for non-scientific research area. We also limit our geographical scope to European regions. Thus, there are still many opportunities to expand this research to a wider range of research fields and geographical areas. Lastly, we adopt a relatively simple metric to measure novelty. We believe our current measure is a fine start to evaluate novelty using keywords in publications but may not be sufficient to capture genuine scientific novelty and may be difficult to consider different aspects of novelty that come from different types of research, e.g., theoretical and computational research or experimental and applied research. In future studies, measurement of novelty should be elaborated to capture novelty more thoroughly and systematically.

## Supporting information

S1 Table Top countries and NUTS2 regions in co-publications. Co-pub is the number of collaborated publications; Sol-pub is the number of non-collaborated publications.
(PDF)

S2 Table Results for each discipline. +p < .1, *p < .05, **p < .01, ***p < .001. ^a^ Collaboration variable.
(PDF)
