# Supplementary material for: Scientific collaboration, research funding, and novelty in scientific knowledge
Source: PLoS One. 2022 Jul 25;17(7):e0271678. doi: 10.1371/journal.pone.0271678 (PMC9312390; doi:10.1371/journal.pone.0271678)
Supplement: S1 Table — Co-pub is the number of collaborated publications; Sol-pub is the number of non-collaborated publications. (PDF) [file pone.0271678.s001.pdf]

S1. Top countries in co-publications

Rank	Top 10 Countries	Co-pub	Sol-pub
**1**	Germany	291,551	509,599
**2**	United Kingdom	269,976	487,607
**3**	France	205,858	370,530
**4**	Italy	171,416	317,156
**5**	Spain	144,046	272,836
**6**	Netherlands	123,197	148,992
**7**	Switzerland	114,959	96,212
**8**	Sweden	89,557	104,054
**9**	Belgium	81,278	73,447
**10**	Austria	74,137	94,484

Co-pub is the number of collaborated publications; Sol-pub is the number of non-collaborated publications.

S1. Top NUTS2 regions in co-publications

Rank	Top 20 NUTS2 Regions		Co-pub	Sol-pub
**1**	FR10	Île de France	147,895	96,938
**2**	UKI3	Inner London – West	112,526	61,807
**3**	ITC4	Lombardia	74,268	39,443
**4**	ES30	Comunidad de Madrid	70,804	47,942
**5**	FR71	Rhône-Alpes	69,752	35,091
**6**	ES51	Cataluña	68,503	49,699
**7**	ITI4	Lazio	68,153	33,798
**8**	DE21	Oberbayern	64,471	29,004
**9**	UKJ1	Berkshire, Buckinghamshire and Oxfordshire	62,287	30,724
**10**	DE30	Berlin	60,002	30,683
**11**	DEA2	Köln	58,130	29,801
**12**	NL33	Zuid-Holland	57,979	27,265
**13**	NL32	Noord-Holland	54,827	21,008
**14**	DE12	Karlsruhe	54,162	24,820
**15**	UKH1	East Anglia	53,186	31,507
**16**	CH04	Zürich	50,733	30,210
**17**	SE11	Stockholm	49,838	27,386
**18**	ITH5	Emilia-Romagna	47,930	21,974
**19**	DK01	Hovedstaden	46,809	30,590
**20**	CH01	Région lémanique	45,937	26,516

Co-pub is the number of collaborated publications; Sol-pub is the number of non-collaborated publications.
