# Supplementary material for: Scientific collaboration, research funding, and novelty in scientific knowledge
Source: PLoS One. 2022 Jul 25;17(7):e0271678. doi: 10.1371/journal.pone.0271678 (PMC9312390; doi:10.1371/journal.pone.0271678)
Supplement: S2 Table — +p < .1, *p < .05, **p < .01, ***p < .001. a Collaboration variable. (PDF) [file pone.0271678.s002.pdf]

S2. Results for Life Science & Biomedicine discipline

	**Dependent variable: Novelty, Life Sciences & Biomedicine**				
	**(1)**	**(2)**	**(3)**	**(4)**	**(5)**
**Intercept**	-1.9221*** (.0035)	-1.9364*** (.0036)	-1.9323*** (.0036)	-1.9307*** (.0036)	-1.9181*** (.0042)
**Country**[a]	-.0062** (.0023)				
**NUTS1**[a]		-.0041* (.0019)			
**NUTS2**[a]			-.0054** (.0016)		
**NUTS3**[a]				-.0058*** (.0014)	
**Institution**[a]					-.0087*** (.0012)
**Funding dummy**	-.0987*** (.0029)	-.0994*** (.0025)	-.0993*** (.0024)	-.0994*** (.0024)	-.0991*** (.0031)
**Col×Funding dummy**	.01*** (.002)	.0092*** (.0014)	.0085*** (.0012)	.008*** (.0011)	.0051*** (.0011)
**# of authors**	-.0031*** (.0006)	-.0031*** (.0007)	-.003*** (.0007)	-.0029*** (.0007)	-.0018*** (.0005)
**# of subjects**	-.0104*** (.0008)	-.0103*** (.0008)	-.0103*** (.0008)	-.0103*** (.0008)	-.0104*** (.0008)
**# of references**	-.0087*** (.0002)	-.0087*** (.0002)	-.0086*** (.0002)	-.0086*** (.0002)	-.0087*** (.0002)
**Country dummy**		.0152*** (.0021)	.0126*** (.002)	.0115*** (.0019)	.0039* (.0018)
**N**	378,955	378,955	378,955	378,955	378,955
**Prob>F**	.0000	.0000	.0000	.0000	.0000
**Vif**	3.11	2.49	2.37	2.29	2.29
**R^2**	.0206	.0208	.0208	.0209	.0216

+p<.1, *p<.05, **p<.01, ***p<.001.

[a] Collaboration variable.

S2. Results for Physical Sciences discipline

	Dependent variable: Novelty, Physical Sciences				
	(1)	(2)	(3)	(4)	(5)
**Intercept**	-1.786*** (.0058)	-1.8268*** (.006)	-1.8321*** (.0058)	-1.8342*** (.0057)	-1.8377*** (.0056)
**Country[a]**	-.0349*** (.0033)				
**NUTS1[a]**		-.0183*** (.0021)			
**NUTS2[a]**			-.0154*** (.0018)		
**NUTS3[a]**				-.0141*** (.0018)	
**Institution[a]**					-.011*** (.0014)
**Funding dummy**	-.1133*** (.0051)	-.1075*** (.004)	-.1057*** (.0038)	-.1051*** (.0038)	-.104*** (.0038)
**Col×Funding dummy**	.0131*** (.0035)	.0081*** (.0021)	.0067*** (.0018)	.0061** (.0018)	.0047** (.0014)
**# of authors**	.0002*** (.0000)	.0003*** (.0000)	.0003*** (.0000)	.0003*** (.0000)	.0004*** (.0000)
**# of subjects**	.008*** (.002)	.0078*** (.002)	.0079*** (.002)	.0079*** (.002)	.008*** (.002)
**# of references**	-.0166*** (.0006)	-.0164*** (.0006)	-.0165*** (.0006)	-.0165*** (.0006)	-.0165*** (.0006)
**Country dummy**		.0283*** (.006)	.0305*** (.0028)	.0315*** (.0027)	.0334*** (.0026)
**N**	182,398	182,398	182,398	182,398	182,398
**Prob>F**	.0000	.0000	.0000	.0000	.0000
**Vif**	4.31	3.99	4.26	4.32	4.62
**R^2**	.0263	.0272	.0272	.0272	.0275

+p<.1, *p<.05, **p<.01, ***p<.001.

[a] Collaboration variable.

S2. Results for Technology discipline

	Dependent variable: Novelty, Technology				
	**(1)**	**(2)**	**(3)**	**(4)**	**(5)**
**Intercept**	-1.6368*** (.0106)	-1.634*** (.0132)	-1.6353*** (.0125)	-1.6341*** (.0118)	-1.6038*** (.0109)
**Country**[a]	-.016* (.0074)				
**NUTS1**[a]		-.0238*** (.0055)			
**NUTS2**[a]			-.0224*** (.005)		
**NUTS3**[a]				-.0221*** (.0045)	
**Institution**[a]					-.0304*** (.0034)
**Funding dummy**	-.1139*** (.0111)	-.1203*** (.009)	-.118*** (.0087)	-.1162*** (.0083)	-.1098*** (.0081)
**Col×Funding dummy**	.008 (.0089)	.0123* (.0061)	.0103+ (.0056)	.0087+ (.0051)	.0046 (.0041)
**# of authors**	-.0002 (.0002)	-.0000 (.0002)	.0001 (.0002)	.0002 (.0002)	.0012*** (.0001)
**# of subjects**	-.0067** (.0026)	-.0066** (.0026)	-.0066* (.0026)	-.0066* (.0026)	-.0068** (.0026)
**# of references**	-.0367*** (.0012)	-.0365*** (.0012)	-.0365*** (.0012)	-.0365*** (.0012)	-.0363*** (.0012)
**Country dummy**		.0102 (.0072)	.0101 (.0069)	.0091 (.0066)	-.0072 (.0061)
**N**	61,449	61,449	61,449	61,449	61,449
**Prob>F**	.0000	.0000	.0000	.0000	.0000
**Vif**	3.90	3.16	3.26	3.16	3.08
**R^2**	.0382	.0389	.0390	.0391	.0416

+p<.1, *p<.05, **p<.01, ***p<.001.

[a] Collaboration variable.
